# Plastic Waste and COVID-19 Incidence Among Hospital Staff After Deescalation in PPE Use

**DOI:** 10.1001/jamanetworkopen.2025.5264

**Published:** 2025-04-15

**Authors:** Stephanie Sutjipto, Aung Hein Aung, Margaret M. L. Soon, Chen Jing, Brenda S. P. Ang, Sapna P. Sadarangani, Kai Wei Chong, Oon Tek Ng, Kalisvar Marimuthu, Wei Yen Lim, Angela Chow, Shawn Vasoo

**Affiliations:** 1 Department of Infectious Diseases, The National Centre for Infectious Diseases, Singapore; 2 Department of Infectious Diseases, Tan Tock Seng Hospital, Singapore; 3 Department of Prevention and Population Medicine, Office of Clinical Epidemiology, Analytics, and Knowledge, Tan Tock Seng Hospital, Singapore; 4 Department of Infection Control, Tan Tock Seng Hospital, Singapore; 5 Lee Kong Chian School of Medicine, Nanyang Technological University, Singapore; 6 Yong Loo Lin School of Medicine, The National University of Singapore, Singapore; 7 Saw Swee Hock School of Public Health, National University of Singapore, Singapore

## Abstract

**Importance:**

In September 2023, the Singapore Ministry of Health revised the national personal protective equipment (PPE) guideline for health care personnel (HCP) attending to suspected or confirmed patients with COVID-19, recommending the use of N95 respirators alone. However, data on the associations between PPE deescalation, staff COVID-19 incidence, and sustainability outcomes are limited.

**Objective:**

To evaluate the associations between PPE deescalation guidelines on staff COVID-19 incidence and the environmental sustainability outcomes of the measure.

**Design, Setting, and Participants:**

This was a retrospective quality improvement study of hospital PPE usage using data from Tan Tock Seng Hospital and the National Centre for Infectious Diseases (NCID), analyzing monthly COVID-19 incidence rates among HCP (all medical staff, allied health care workers, ancillary staff, and administrative staff from the hospital campus) and in the community over 12 months before and after PPE deescalation measures (October 2021 to September 2022 and October 2022 to September 2023).

**Main Outcomes and Measures:**

COVID-19 incidence rates among HCP and in the community; economic and environmental outcomes of removing single-use gowns from routine COVID-19 care, including reductions in carbon footprint, plastic waste generation, and cost savings.

**Results:**

The mean (SD) monthly number of HCP was 10 774 (79) (range, 10 636-10 891) preimplementation and 11 099 (200) (range, 10 864-11 449) postimplementation. Our analysis revealed PPE deescalation was not associated with an increase in monthly COVID-19 infections among hospital staff, with the trends aligning with population infection rates. The median (IQR) staff COVID-19 infection rate relative to the community COVID-19 infection rate was 2.6 (1.9-3.6) preimplementation compared with 1.5 (0.9-3.1) postimplementation. An estimated 4 gowns per patient-day were saved, totaling 440 532 gowns over 12 months. This equated to an estimated reduction in health care costs by SGD 453 748 (approximately USD 333 970) and reductions of 398 681.46 kg carbon dioxide equivalent in carbon emissions and 66 080 kg of plastic waste.

**Conclusions and Relevance:**

This quality improvement study of hospital PPE usage observed that the national PPE deescalation guidelines corresponded with the reductions in protective gown use, associated costs, carbon footprint, and plastic waste generation with no apparent compromise to staff safety and health.

## Key Points

**Question:**

Following PPE deescalation in Singapore, what was the incidence of COVID-19 among staff and how did the amount of plastic waste change?

**Findings:**

This quality improvement study analyzed PPE usage and staff COVID-19 incidence before and after PPE deescalation. Staff-to-community COVID-19 rates were comparable with median community rates of 2.6 preimplementation and 1.5 postimplementation; the implementation was associated with a 12-month reduction of 440 532 gowns, 398 681.46 kg carbon dioxide equivalent in carbon footprint, 66 080 kg plastic waste, and approximately SGD 453 748 (approximately USD 333 970) in health care costs.

**Meaning:**

These findings suggest PPE deescalation to N95 respirators alone ensured staff safety, promoted environmental sustainability, and reduced associated costs.

## Teaser

This quality improvement study evaluates whether personal protective equipment (PPE) deescalation guidelines in Singapore was associated with staff COVID-19 incidence as well as the environmental sustainability outcomes of PPE deescalation.

## Introduction

At the onset of the COVID-19 pandemic, the World Health Organization (WHO) provided guidelines for personal protective equipment (PPE) for health care personnel (HCP) caring for patients with SARS-CoV-2 infections. These included gowns, face shields, gloves, and medical masks, with the use of respirators during aerosol-generating procedures.^1,2^ By early 2023, the WHO declared the pandemic over, recommending a shift from emergency responses to standard infectious disease management.^3^ However, the PPE guidance from public health authorities remains largely unchanged. The US Centers for Disease Control and Prevention continues to recommend National Institute for Occupational Safety and Health–approved particulate respirators (N95 respirators or higher), gowns, gloves, and eye protection,^4,5^ while the European Centre for Disease Prevention and Control recommends respirators (with extended use or medical masks in PPE shortages), along with gowns and gloves when there is a risk of bodily fluids contact.^6^

On September 27, 2022, the Singapore Ministry of Health revised PPE guidance for HCP attending to suspected or confirmed patients with COVID-19, recommending the use of N95 respirators alone. This eliminated routine use of gowns, face shields or eye protection, and gloves for routine COVID-19 care. The decision considered the following factors: (1) Omicron and its subvariants were associated with milder illness, (2) there was substantial population hybrid-immunity via COVID-19 vaccination and/or natural infection, and (3) local studies found no significant PPE contamination by the virus, even after multiple HCP-patient interactions. Findings of substantial environmental contamination by SARS-CoV-2 via respiratory droplets highlight this as a potential transmission medium instead.^5,7,8^

This report evaluated the associations between national PPE deescalation and COVID-19 infection rates among HCP at our hospital, estimated the PPE and cost savings from eliminating single-use gowns from standard COVID-19 care, and examined the sustainability implications. Before the pandemic, the global health care sector contributed 4.4% of net emissions, equivalent to 2 gigatons of carbon dioxide (CO_2_) annually.^9^ The pandemic triggered a dramatic surge in health care waste; in Wuhan, China, medical waste increased by nearly 400%, from 50 tons per day prepandemic to 247 tons per day at the peak of the pandemic.^10^ Sterilization plants in Madrid, Spain, operated at full capacity processing 50 tons per day, while Catalonia managed 1200 tons of health care waste between mid-March to mid-April 2020, a 300% to 350% rise from the baseline of 275 tons per month.^11^ In Bangladesh, approximately 14 500 tons of health care waste were generated at the pandemic’s peak.^12^

The pandemic also intensified environmental pollution, with PPE disintegrating into microplastics and microfibers, which persist in ecosystems for years, accumulating in river water and sediments.^13,14^ Following PPE deescalation in Singapore, we examined the incidence of COVID-19 among staff and environmental sustainability changes in terms of reduction in the carbon footprint and plastic waste achieved.

## Methods

This retrospective review of hospital PPE usage was conducted as part of a quality improvement initiative. Ethical approval was not required, as anonymized data collection was performed as part of an intrahospital PPE usage audit for quality improvement purposes. Hospital-level approval for the use of unidentifiable hospital operational data for publication was obtained. Anonymized extracted data were stored on secure servers. The study adhered to the Standards for Quality Improvement Reporting Excellence (SQUIRE) reporting guidelines.^15^

### Statistical Analysis

The National Centre for Infectious Diseases (NCID) is a medical center located adjacent to the Tan Tock Seng Hospital (TTSH) on the HealthCity Novena campus in Singapore. The campus has a combined capacity of about 2000 beds, including 300 beds in NCID, and employs approximately 11 000 staff. During most of the pandemic phases, NCID was the major center providing tertiary care for about 25% to 30% of inpatients with COVID-19 in Singapore. TTSH, through its emergency department, was the main conduit for admissions of patients with COVID-19 to NCID and intermittently housed patients with COVID-19 during disease surges. For our analysis of COVID-19 incidence among HCP, we included all medical staff (physicians, nurses, and nursing assistants), allied health care workers, ancillary staff, and administrative personnel working in both NCID and TTSH.

To determine the monthly incidence of COVID-19 among HCP, we analyzed staff health databases for the 12 months before (October 2021 to September 2022) and after (October 2022 to September 2023) the PPE deescalation measures. The 12-month timeframes were chosen to evaluate the outcomes of COVID-19 PPE recommendations across various pandemic phases and surges. Our objective was to investigate any shift or increase in potential nosocomial COVID-19 acquisition risks among HCP following the national PPE deescalation.

The monthly number of HCP who had COVID-19 infections was determined by TTSH’s surveillance database, which included (1) staff illness reports from the hospital’s Staff Health Surveillance System; (2) COVID-19 illness reports from online reporting platform, FormSG; and (3) hospital laboratory data of COVID-19 polymerase chain reaction and rapid antigen tests. These monthly HCP numbers were obtained from the hospital’s administrative database. The monthly COVID-19 infection rates among HCP were determined by calculating the proportion of infections among HCP to the total number of HCP for each respective period. The monthly COVID-19 infection rates among HCP were additionally compared with the reported COVID-19 incidence in the Singapore general population from the country-reported data to the WHO.^16,17^

We also calculated the rate of community COVID-19 cases by using the yearly population data from Statistics Singapore^18^ and compared it with the COVID-19 infection rate among HCP. Wilcoxon rank-sum test was applied to compare the median of rates at different time points before and after the PPE change. This comparative analysis aimed to consider the fluctuating COVID-19 community infection pressure, particularly during surges attributable to the emergence of various SARS-CoV-2 variants. An interrupted time series multivariable regression analysis (adjusted for staff and community population sizes) was performed to compare the 12-month baseline incidence of COVID-19 in HCP before the change in PPE (time series 1) and the 12-month post-PPE change period (time series 2) to assess the correlation between the PPE change and HCP COVID-19 incidence.

We excluded TTSH in estimating gown savings as gown usage at TTSH was primarily for non–COVID-19 indications (eg, for multidrug resistant organisms contact precaution). The number of protective gowns used at the NCID during the 12 months before and after PPE deescalation was obtained from the hospital’s inventory records. The daily number of admitted patients to NCID and their length of stay were obtained from the hospital’s bed occupancy data to tabulate the total patient-days for each month during 24-month study periods. The *t* test was performed to evaluate the difference in the mean number of gowns used per patient-day during the 2 periods.

The protective gown usage was then divided over the mean patient-days of each month to obtain the number of gowns used per patient-day in the hospital. Using the preimplementation number of gowns used per patient-day, we estimated the amount of gown usage in the following 12 months if no PPE deescalation was implemented. The number of gowns saved with PPE deescalation was then calculated by subtracting the actual gowns used in the postimplementation period from the expected number of gowns used without PPE deescalation.

Due to the vast differences in the intensity of medical and nursing care requirements of patients with COVID-19 admitted to the general wards (GW) and intensive care units (ICU), data were further separated to reflect usage in the 2 areas. We did not estimate the change in the use of gloves pre- and postimplementation of the PPE deescalation as this was less specific to COVID-19 care.

The carbon footprint is one of the common measurement factors used to estimate a product’s or activity’s environmental impact and greenhouse gas emissions resulting in climate change. The CO_2_ equivalent (CO_2_e) represents the total amount of direct and indirect greenhouse gas emissions caused by a product or event.^19^ The CO_2_e for a single-use protective gown was previously estimated in earlier literature^20^ to be approximately 905 g CO_2_e (ie, the amount of CO_2_e impact on global warming of a single-use protective gown; calculated based on a combination of emissions burden from the life cycle inventory assessment and various global environmental impacts). CO_2_e is a unit of measurement that indicates the global warming potential of various greenhouse gases and is useful for comparisons.^21^ The total carbon footprint saved by eliminating the use of single-use protective gowns for routine COVID-19 care was calculated based on the number of gowns saved with the PPE deescalation multiplied by 905 g CO_2_e.

Protective gowns and the plastic packing unit supplied to the hospital were weighed using a digital scale, and the total weight was then divided by the number of protective gowns per package to obtain the plastic weight of each protective gown. Plastic waste generation saved by the PPE deescalation was then calculated using the estimated number of gowns saved multiplied by the plastic weight of a protective gown.

The NCID uses fluid-repellent, long-sleeve polypropylene-polyethylene isolation gowns with wrist cuffs for the care of patients with COVID-19. There were 3 types of protective gowns used in the hospital for care of patients with COVID-19 during the studied periods (hospital codes: 005A, 002A, and 003C). The total cost saved by removing routine protective gowns from COVID-19 care was calculated using the estimated number of gowns saved multiplied by the cost of each gown.

Regression coefficients with 95% CIs are presented. A 2-tailed *P* value less than .05 was considered to be statistically significant. All statistical analyses were performed using Stata/SE-13.0 (StataCorp).

## Results

### Incidence of COVID-19 Infection Among HCP

Due to various surges in COVID-19 cases since the onset of the pandemic, the total number of HCP has fluctuated monthly, influenced by augmented manpower and staff turnover. The mean (SD) monthly number of HCP in the preimplementation period was 10 774 (79) personnel (range, 10 636-10 891), and the mean monthly number of HCP in the postimplementation period was 11 099 (200) personnel (range, 10 864-11 449).

**Figure 1.**
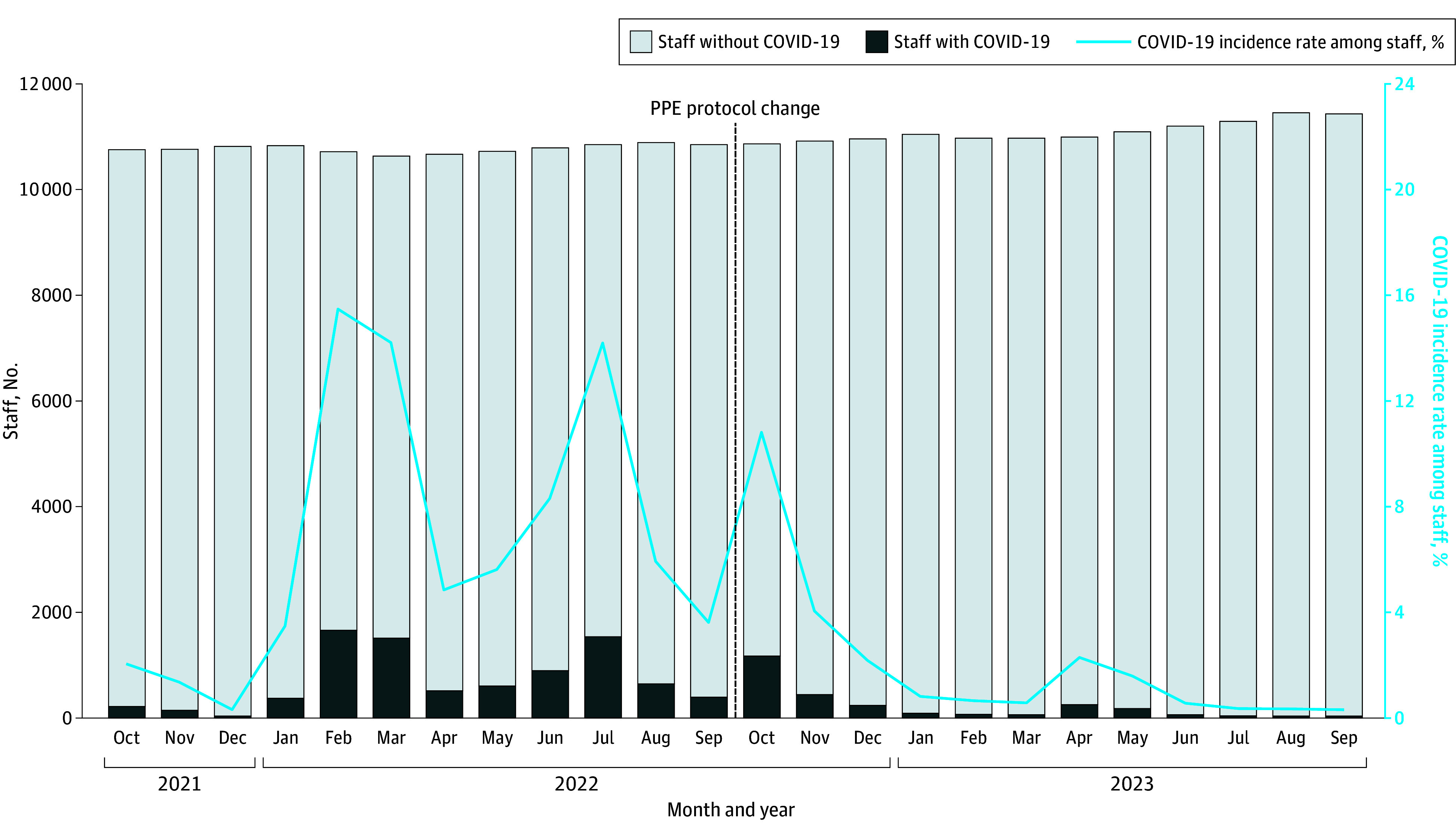
Monthly Incidence of COVID-19 Among Staff, 12 Months Before and After the Change in the National Personal Protective Equipment (PPE) Protocol

**Figure 2.**
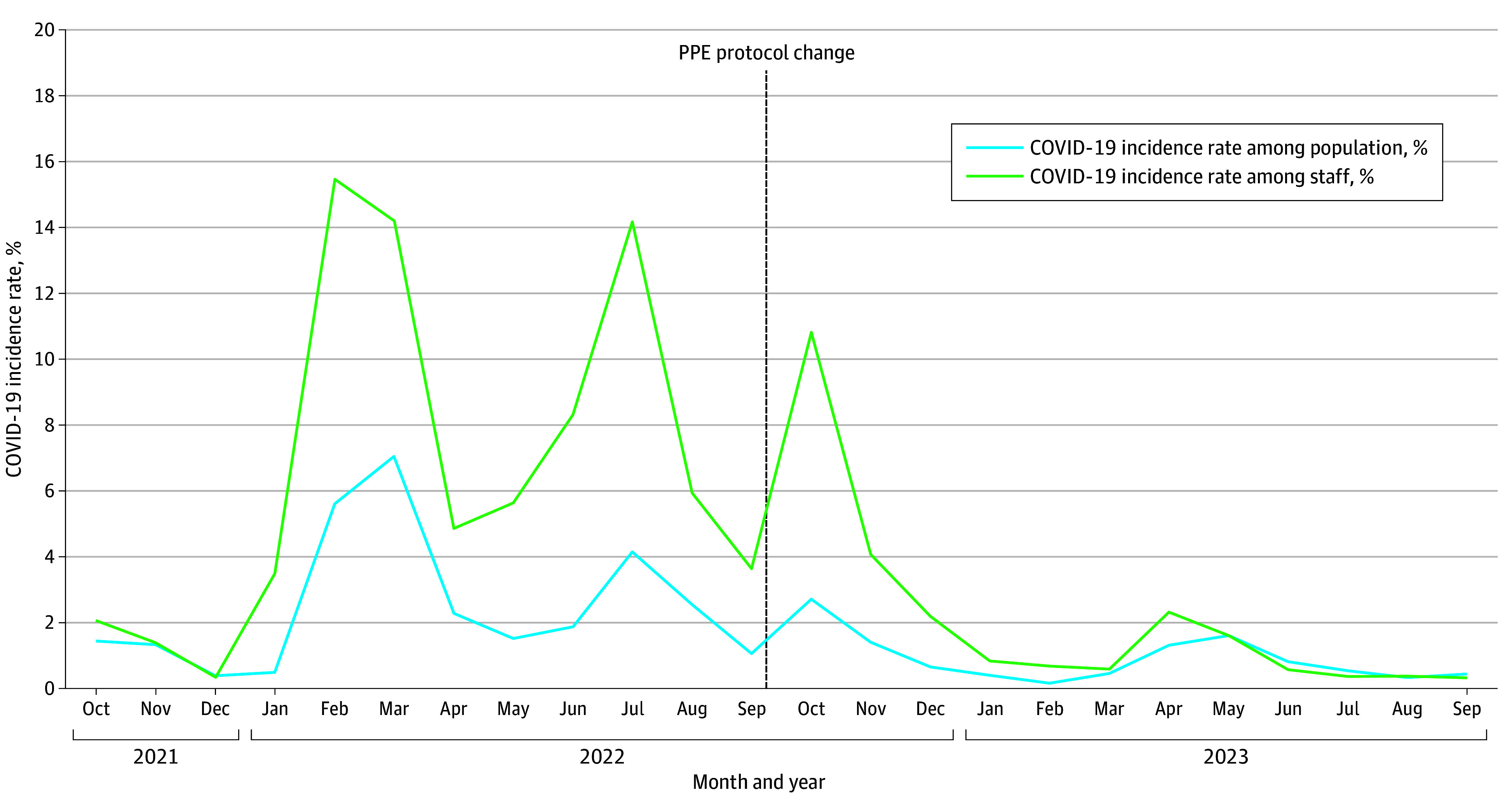
Monthly Rates of COVID-19 in the Community and Among Staff, 12 Months Before and After the Change in the National Personal Protective Equipment (PPE) Protocol

The mean (SD) monthly incidence of COVID-19 among staff in the preimplementation period was 6.6% (5.3); the highest incidence was in February 2022 at 15.5% (median [IQR], 5.2 [3.1%-9.8%]). In the postimplementation period, the mean (SD) incidence of COVID-19 among staff was 2.1% (3.0), with the highest incidence in October 2022 at 10.8% (median [IQR], 0.7% [0.5%-2.2%]) (Figure 1). The 4 peaks in COVID-19 incidence among HCP closely mirrored those observed in the community, including the outlier month of October 2022 of the postimplementation period (Figure 2). Staff had a median (IQR) of 2.6 (1.9-3.6) times higher rate than the community during the period before the PPE protocol changed. The reported rate of COVID-19 infection among staff is expected to be higher than that of the community due to nationally mandated rostered routine testing for SARS-CoV-2 for HCP from May 2021 to March 2022, followed by enhanced hospital surveillance and staff health reporting thereafter. This proportion remained unchanged after the PPE change, with the median (IQR) staff COVID-19 infection rate 1.5 (0.9-3.1) times higher than the community rate postimplementation. The implementation of PPE deescalation did not appear to be associated with the rate of COVID-19 incidence among HCP, with the overall trend continuing to parallel the community’s (Figure 2).

An interrupted time series multivariable regression analysis (adjusted for staff and community population sizes) to compare the incidence of COVID-19 in HCP in the 12 months before the change in PPE measures (time series 1), and in the 12 months after the PPE change (time series 2) showed no significant association between PPE deescalation and COVID-19 incidence in HCP. Instead, the incidence of COVID-19 in HCP was significantly and positively associated with the incidence of COVID-19 in the community (*r* = 0.01, *P* < .001) (Table 1).

**Table 1.** Interrupted Time Series Multivariable Regression Analysis to Explore the Association Between Change in the National Personal Protective Equipment (PPE) Protocol and Community COVID-19 Pressure on the Incidence of COVID-19 Among Staff^a^

Variable	Coefficient (95% CI)	*P* value
Monthly HCP No.	1.05 (−0.28 to 2.37)	.11
Population size	0.00 (−0.00 to 0.00)	.82
Monthly community COVID-19 No.	0.01 (0.00 to 0.01)	<.001
Implementation of PPE changes	22.26 (−320.17 to 364.68)	.89
Time series of the whole study period (October 1, 2021 to September 30, 2023)	20.53 (−22.65 to 64.71)	.34
Time series after PPE change (October 1, 2022 to September 30, 2023)	−111.81 (−183.00 to −40.62)	.004

Abbreviation: HCP, health care personnel.

^a^ This analysis compared the 12 months’ time points before and after the change in PPE protocol with adjusted HCP and community population sizes.

### Estimation of Single-Use Protective Gown Consumption

The number of protective gowns used per patient-day at NCID during the 12 months before and after PPE deescalation is summarized in Table 2. With the implementation of PPE deescalation, the number of gowns per patient-day was reduced by 10.6 (95% CI, 6.67-14.59) in the GW, 20.9 (95% CI, 10.52-31.27) in the ICU, and 11.04 (95% CI, 7.68-15.39) overall. When comparing the actual number of gowns used with the projected usage if PPE deescalation measures had not been implemented, the estimated number of gowns saved during the postimplementation period was 407 405 in the GW, 33 127 in the ICU, and 440 532 gowns in total (Table 2).

**Table 2.** Mean Gowns Per Patient-Day Used at National Centre for Infectious Diseases During the 12 Months Before and After Implementation of Personal Protective Equipment (PPE) Deescalation Protocol

Location	Mean gowns per patient-day (SD)		Reduction of gowns used per patient-day after PPE deescalation (estimated 95% CI)	Total patient-days in postimplementation period (October 2022 and September 2023)	Estimated No. of gowns saved after PPE deescalation
	Preimplementation period (October 2021 to September 2022)	Postimplementation period (October 2022 to September 2023)			
General wards	12.09 (6.54)	1.46 (1.00)	10.63 (6.67-14.59)	38 326	407 405
Intensive care unit	40.19 (16.55)	19.29 (5.14)	20.90 (10.52-31.27)	1585	33 127
Total	13.22 (6.38)	2.17 (0.90)	11.04 (7.68-15.39)	39 911	440 532

### The Carbon Footprint Values of Single-Use Protective Gowns

Assuming the CO_2_e of each gown to be 905 g CO_2_e,^20^ the total amount of carbon footprint reduction from the 440 532 gowns saved after the PPE deescalation was approximately 398 681 460 g CO_2_e (ie, 398 681.46 kg CO_2_e). This is equivalent to approximately 9.99 kg CO_2_e of carbon footprint saved per patient-day in the postimplementation period.

### Plastic Waste Generation of Single-Use PPE

We assessed the weight of a pack of 10 protective gowns used for COVID-19 care, including the plastic packaging supplied to the hospital, and ascertained it to be 1.5 kg per package. This equates to approximately 0.15 kg of plastic weight per gown. Therefore, the total reduction in plastic waste generation from saving 440 532 gowns after the PPE deescalation was approximately 66 080 kg of plastic over 12 months, or 1.65 kg of plastic saved per patient-day in the postimplementation period.

### Cost Savings

The cost of a protective gown was SGD 1.03 (approximately USD 0.76). With approximately 440 532 gowns saved by the PPE deescalation, we estimated a reduction in health care costs by a total of SGD 453 748 (approximately USD 333 970) in the 12 months postimplementation, or SGD 11.40 (USD 8.41) saved per patient-day from eliminating gown usage in routine COVID-19 care.

## Discussion

In Singapore, the transition from a pandemic to an endemic approach began in September 2022. This included updated health advisories and PPE recommendations, limiting PPE to N95 respirators for routine care for patients with COVID-19.^8^ A recent systematic review found that while N95 and surgical masks significantly protect HCP against SARS-CoV-2, the effectiveness of gloves, gowns, and face shields is less clear, with some evidence suggesting gloves may increase infection risk by fostering a false sense of security.^22^ Additionally, subanalysis of data on gowns and gloves usage revealed no significant changes in COVID-19 infection rates among HCP.^23^

Our review of staff COVID-19 infection before and after PPE deescalation did not find a significant increase in HCP COVID-19 incidence, with the staff’s trend closely mirroring that of the community. The findings suggest that removing protective gowns and eye shields from routine COVID-19 care did not significantly alter transmission risks to HCP. PPE deescalation was also associated with a reduction of approximately 11.04 gowns per patient-day at NCID, leading to an estimated 440 532 gowns saved over the subsequent 12 months and health care cost savings of approximately SGD 453 748. Additionally, the reduction in gown usage contributed to a decrease in the hospital’s carbon footprint. Using the conversion factors provided by the Environmental Protection Agency, 398 681 kg CO2e equals the emissions from 169 817 liters of gasoline consumed. It also equals the greenhouse gas emissions avoided by recycling 138 tons (approximately 125 191 kg) of waste instead of landfilling and is equivalent to the carbon sequestered by 6592 tree seedlings grown for 10 years.^24^

According to the manufacturer’s information,^25^ the Medtecs CoverU Disposable Isolation Gown (IL-4036YK) commonly used in our hospital for COVID-19 care consists of 55% polypropylene and 45% nonwoven polyethylene. The disposal of polypropylene PPE waste during the pandemic has led to an environmental hazard, with over 4 million metric tons of PPE waste and microplastic byproducts entering the environment since the pandemic’s onset.^26^ Polypropylene, resistant to biological degradation, can remain in the natural environment for up to 450 years.^26^ Polyethylene, known for its durability and low permeability, is nonbiodegradable, making complete degradation under uncontrolled conditions challenging.^27^

Efforts to reduce carbon emissions and plastic waste from the health care sector are vital to highlight the sector’s responsibility in promoting sustainability. For instance, recent evidence suggests that contact precautions may not significantly reduce transmission rates of pathogens such as methicillin-resistant *Staphylococcus aureus* and vancomycin-resistant *Enterococcus* in institutions with robust infection prevention practices, including high hand hygiene compliance and other horizontal infection prevention strategies.^28^ Furthermore, eliminating unnecessary precautions can improve health care worker–patient interactions and reduce negative psychological impacts on patients without compromising infection control outcomes.^28^ Exploring alternatives to single-use plastic gowns, such as reusable gowns or those made from more sustainable materials, is essential. However, these approaches must be carefully balanced to ensure both staff safety and effective infection control.

Singapore was among the first countries to implement PPE deescalation for COVID-19. PPE deescalation was associated with substantial reductions in protective gown usage, significant cost savings, and notable environmental benefits without any corresponding increase in COVID-19 HCP infections above the community infection rate. Given the widespread availability of effective vaccines, decent levels of herd immunity, and the generally milder illness with current circulating Omicron variants, it is timely that health authorities reexamine their PPE recommendations for COVID-19.

### Limitations

The main limitation of our study arose from attributing HCP COVID-19 incidence to nosocomial vs community acquisition, as COVID-19 was considered endemic in the community. Therefore, we relied on publicly available national data to ascertain COVID-19 community infection rates, and to compare these with HCP infection rates. Underreporting may have occurred in HCP data due to the cessation of mandated testing after March 2022. In our manuscript, we could not directly attribute all gown usage at NCID solely to COVID-19, and the estimated number of gowns saved could vary depending on COVID-19 infection surges. Consequently, these estimates may overstate or understate the carbon footprint calculations. It is also important to note that protective gowns continued to be used for non–COVID-19 reasons after PPE deescalation, particularly for patients with multidrug resistant organisms. Additionally, local and regional data on the life cycle analysis for calculating the carbon footprint was unavailable at present, so we relied on previously published estimates. Despite this limitation, we believe these findings reflect reliable insights into the change in carbon footprint outcomes from PPE deescalation at our center.

## Conclusions

Our findings underscore the importance of ongoing evaluation and optimization of PPE protocols for infectious diseases, considering the biological basis of disease transmission route(s), transmissibility, and availability of countermeasures. Evidence-based PPE rationalization helps to promote sustainable health care practices that repeat benefits in environmental sustainability and costs without compromising safety. Climate action demands urgent evidence and a critical reevaluation of recommended PPE for COVID-19 and other infectious diseases.

## Notes

Supplement.
Data Sharing Statement
