# Supplementary material for: Plastic Waste and COVID-19 Incidence Among Hospital Staff After Deescalation in PPE Use
Source: JAMA Netw Open. 2025 Apr 15;8(4):e255264. doi: 10.1001/jamanetworkopen.2025.5264 (PMC12000988; doi:10.1001/jamanetworkopen.2025.5264)
Supplement: Supplement. — Data Sharing Statement [file jamanetwopen-e255264-s001.pdf]

Data Sharing Statement

Sutjipto. Plastic Waste and COVID-19 Incidence Among Hospital Staff After Deescalation in PPE Use. *JAMA Netw Open*. Published April 15, 2025.
doi:10.1001/jamanetworkopen.2025.5264

Data
Data available: No
